# Accelerometer-measured stepping cadence patterns in Korean adults: an analysis of data from the 2014-2015 Korea National Health and Nutrition Examination Survey

**DOI:** 10.4178/epih.e2021056

**Published:** 2021-08-17

**Authors:** Geon Hui Kim, Hoyong Sung, Yeun Ryu, Jungjun Lim, Joon-Sik Kim, Hak Kyun Kim, Yeon Soo Kim

**Affiliations:** 1 Department of Physical Education, College of Education, Seoul National University, Seoul, Korea; 2 Institute of Sports Science, Seoul National University, Seoul, Korea

**Keywords:** Walking, Exercise, Sedentary Behavior, Accelerometry

## Abstract

**OBJECTIVES:**

The aim of this study was to identify the stepping cadence patterns in Korean adults by using objectively measured accelerometer data to analyze the time spent in each cadence category by sex and age.

**METHODS:**

During the 2014-2015 Korean National Health and Nutrition Examination Survey, 1,703 males and females aged 19-64 years provided at least 1 valid day of data (wearing an accelerometer ≥ 10 hr/d). The mean cumulative time and percentage per day in 8 cadence categories (0, 1-19, 20-29, 30-59, 60-79, 80-99, 100-119, and ≥ 120 steps/min) by sex and age group were calculated.

**RESULTS:**

Cumulative time and percentage per day decreased across the incremental cadence categories. Participants spent 360.08± 2.56 min/d in the non-movement cadence category and 361.50± 2.28 min/d in the incidental movement cadence category. However, they spent only about 18.1 min/d (2.1%) at ≥ 100 steps/min. Males spent significantly more time in the cadence categories of sporadic movement, purposeful steps, slow walking, and medium walking, but the other categories, except for brisk walking, had higher values in females (p< 0.001). The older age group spent less time in non-movement cadence categories, and the youngest and oldest groups spent more time at a higher cadence (≥ 100 steps/min) than the other age groups. Similar patterns were found in a subgroup analysis by sex.

**CONCLUSIONS:**

Korean adults spent most of their time at a low cadence and only a few minutes at a high cadence (≥100 steps/min); this trend was consistent across sex and age groups.

## INTRODUCTION

Step amount is a common and simple indicator of physical activity (PA) during free-living behavior [1]. It is a well-known prognostic factor for mortality, cardiovascular disease, and metabolic disorders such as type 2 diabetes [2,3]. Thus, step amount is frequently used in PA prescriptions; however, step amount does not include information on the intensity of step-based PA.

The intensity of PA, which refers to the rate of energy expended during PA and is commonly expressed as metabolic equivalents (METs), is an essential factor to consider when prescribing PA [1]. The intensity of PA has shown significant associations with the risk of mortality and chronic diseases. In particular, a higher proportion of vigorous PA was found to be associated with lower mortality risk and a low prevalence of metabolic diseases [4,5]. Accordingly, most PA guidelines provide criteria for the intensity of PA [1,6].

Stepping cadence, which is defined in terms of steps per minute, has been used in several studies as an indicator of intensity during free-living activity. A strong correlation (0.94) was found between cadence and METs in a laboratory study [7]. A cadence of 100 steps/min corresponds to 3 METs (moderate intensity), and a cadence of 130 steps/min is equivalent to 6 METs (vigorous intensity) [8-10]. Thus, stepping cadence can be appropriate as a way to assess the intensity of step-based PA during free-living behavior.

In previous studies, the accelerometer-measured free-living stepping cadence patterns in Unite States adults and children were examined using data from the National Health and Nutrition Examination Survey [11-15]. Stepping cadence was associated with cardiometabolic risk factors [16,17]. However, despite this significant relationship, to our knowledge, stepping cadence patterns in the Korean population have not been reported to date. Therefore, this study aimed to identify the stepping cadence patterns in Korean adults by sex and age groups using Korea National Health and Nutrition Examination Survey (KNHANES) data, a large representative dataset of general Korean adults.

## MATERIALS AND METHODS

### Data source

KNHANES has been conducted annually by the Korea Centers for Disease Control (now known as the Korea Disease Control and Prevention Agency) to analyze the health and nutrition status of Koreans aged 19 to 64 since 1998. An accelerometer (Actigraph GT3X+; Actigraph, Pensacola, FL, USA) was used to collect objective PA data in 2014. In the 2014 and 2015 KNHANES, 1,827 males and females agreed to wear an accelerometer for 7 consecutive days. They were asked to wear the device on their hip using an elastic band and instructed to remove it only at bedtime and during water-based activities such as swimming and bathing. Sixty participants were excluded because the accelerometer was missing (n= 9), not worn (n= 47), or broken (n= 3), or because they had invalid data (n= 1). Thus, 1,767 adults were included in the public accelerometer data set.

### Data treatment

The National Cancer Institute’s SAS code was applied to the accelerometer data cleansing process [18]. A valid day was defined as at least ≥ 10 hr/d of wearing time, and non-wearing time was defined as ≥ 60 minutes of consecutive zero activity counts, allowing ≤ 2 minutes of counts between 0 to 100. Among 1,767 participants in the public data set, 63 participants without 1 valid day were excluded. In addition, 1 participant who was missing height and weight data was excluded from the analysis. Finally, 1,703 participants with at least 1 valid day of data were included in the analysis.

Accelerometer data were recorded in 1-minute epochs. Daily minute-by-minute step data were summed by minutes for each cadence category and were averaged across valid days within individuals. Step cadence was defined as step counts per minute and categorized using the following criteria used by Tudor-Locke et al. [15]: non-movement (0 steps/min), incidental movement (1-19 steps/min), sporadic movement (20-39 steps/min), purposeful steps (40-59 steps/min), slow walking (60-79 steps/min), medium walking (80-99 steps/min), brisk walking (100-119 steps/min), and faster locomotion (≥ 120 steps/min). Stepping cadence values were examined by cadence category across sex and age groups (19-29, 30-39, 40-49, 50-59, and 60-64 years).

Demographic variables such as sex, age, height, weight, and body mass index (BMI) were included in this study. BMI was calculated as weight divided by height squared. BMI status was classified as underweight (< 18.5 kg/m^2^), normal weight (18.5-22.9 kg/m^2^), overweight (23.0-24.9 kg/m^2^), and obesity (≥ 25.0 kg/m^2^) based on the World Health Organization cut-points for Asian populations [19].

### Statistical analysis

Participants’ demographic characteristics across sex and age groups were expressed as means and standard errors (SE) for continuous variables and counts and percentages for categorical variables. In addition, descriptive statistics of each cadence category were presented as the mean and SE of cumulative time and percentage, which was calculated as the cumulative time divided by the total wearing time, according to sex and age groups. The mean differences of cumulative time and percentage in each cadence category between males and females were examined using the independent t-test. In addition, linear trends in the time of each cadence category across age groups were assessed.

Statistical significance was set a level of 0.05. All statistical analyses were performed using R version 4.0.3 (https://cran.r-project.org/).

### Ethics statement

The Institutional Review Board (IRB) of Seoul National University (IRB No. E2105/001-005) approved this study protocol, and the requirement to obtain informed consent was waived by the board.

## RESULTS

A total of 1,703 Korean adults wore an accelerometer for 13.80±1.58 hr/d and had 5.39± 1.76 valid days on which they wore the accelerometer for at least 10 hours. The demographic characteristics of the overall, and for each sex and age group are shown in Table 1. Although the mean daily step count of all participants was about 7,968.68± 3,723.02 steps/d, there was a significant difference between sex (p< 0.001). Moreover, older groups tended to have a higher mean daily step count (p for trend < 0.001).

Table 2 presents the descriptive statistics of time spent in each cadence category overall and by sex. Participants accumulated 360.08± 2.56 min/d at non-movement category and spent 361.50±2.28 min/d in the incidental movement category (1-19 steps/min). The total time spent in the non-movement and incidental movement cadence categories was about 12 hr/d, which contributed to almost 87% of the wearing time. Cumulative time and percentage per day showed low values across the incremental cadence category. No significant difference was found in time spent at a non-movement cadence between males and females (p= 0.625). Males significantly spent more time in the cadence categories of sporadic movement, purposeful steps, slow walking, and medium walking; however, the other categories, except for brisk walking, had higher values in females (p< 0.001).

Table 3 shows the time spent in each cadence category across age groups. While the youngest group (aged 19-29) spent the most time (6.7 hr/d) at non-movement cadence, the oldest group spent the least time (5.6 hr/d) at the same cadence level. On the contrary, the time spent at incidental movement cadence was highest in the oldest group (aged 60-64) and lowest in the youngest age group. Significant linear trends were found in time spent and its percentages across age groups, except for the cadence categories of purposeful steps and slow walking (p< 0.001).

**Figure 1.**
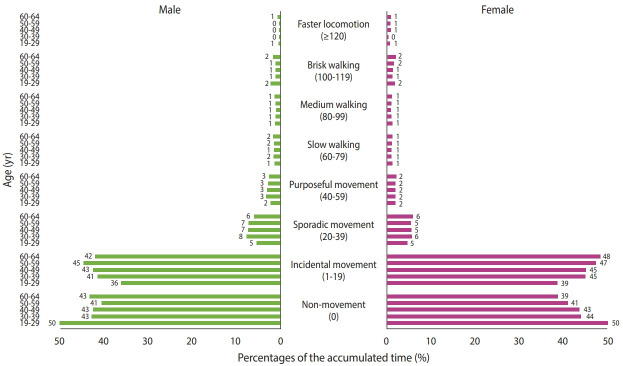
The percentage of time spent in each cadence category (unit: steps/min) across age groups by sex. The percentage of cumulative time in each cadence across age groups in male (left) and female (right).

The percentage of spent time relative to total wearing time in each cadence category by sex and age groups is presented in Figure 1. Regardless of age and sex groups, the participants spent the most time in the non-movement or incidental movement categories. Only approximately 10% of the participants spent relatively more time in the fast cadence category.

## DISCUSSION

This study aimed to identify the descriptive statistics of accelerometer-measured free-living stepping cadence in Korean adults through an analysis of KNHANES data, which are representative of the Korean population. For all participants, most of the time (about 12 hr/d, 87% of wear time) was spent in the cadence categories of non-movement (0 steps/min) and incidental movement (1-19 steps/min). The cumulative time at a higher cadence corresponding to walking or locomotion (≥ 60 steps/min) was only about 30 minutes (4.0% of wearing time). The cumulative time and percentage of most stepping cadence categories differed across sex and age groups, whereas the linear trends in cumulative time and percentage of time spent were similar across cadence categories in most groups.

Consistent with our results, linear trends have been found in cumulative time and percentage across incremental cadence categories in previous studies. However, different results were found for cumulative time and percentage per day in each cadence category in different countries. Tudor-Locke et al. [15] reported stepping cadence patterns in Unite States adults using the 2005-2006 National Health and Nutrition Examination Survey. A total of 3,744 United States adults spent about 4.8 hr/d (34%) at a nonmovement cadence and about 6.3 hr/d (46%) at an incidental movement cadence. In contrast, Summer et al. [20] reported that, in a study of 713 multi-ethnic Asian individuals (Chinese, Malay, and Indian), the mean cumulative time was approximately 7.9 hr/d (53%) at non-movement cadence and 5.4 hr/d (36%) at incidental movement cadence. In our samples, KNHANES participants spent approximately 6 hr/d (43%) in the non-movement category and 6 hr/d (44%) in the incidental movement cadence category. Thus, Asians are likely to spend more time in non-movement categories than Americans based on the results. For the cadence categories of more than 20 steps/min, the cumulative time and proportion were similar to those of other Asians and Koreans. However, Americans spent more time (both in absolute and relative terms) at a lower cadence (< 80 steps/min), whereas they showed slightly lower values at a higher cadence.

Non-movement cadence refers to no ambulation for a period of time, which indicates sedentary behavior [21]. Based on the current study results, the average sitting time of Korean adults was about 6 hr/d, which is lower than that measured by both the self-reported questionnaire and the tri-axial accelerometer. Individuals in the same KNHANES sample reported a sitting time of about 7.7 hr/d using a self-report PA questionnaire, and their sitting time was approximately 8.3 hr/d as measured by a tri-axial accelerometer [22]. Similarly, in the same United States adult sample, sedentary time measured by the accelerometer activity count was 1.7 times higher than sedentary time calculated in terms of step cadence [23]. This difference may be attributed to the relatively strict classification criteria for sedentary behavior based on cadence. In the current study, non-movement cadence was defined as all minutes corresponding to 0 steps. Thus, 1 or 2 isolated steps were classified as a cadence of incidental movement. Conversely, sedentary behavior was defined as below 100 activity counts per minute in an activity count-based study. Wong et al. [21] reported that a threshold of 100 counts per minute could only distinguish 88.1% of sedentary behavior defined as 0 steps/min in adults. Thus, it is unclear which criterion more accurately classifies sedentary behavior, but it should be noted that the sedentary time may be undervalued when evaluated based on stepping cadence compared to if other methods are used.

With a high percentage of sedentary time, the predominant cadence patterns in our sample were at a low cadence (<40 steps/min). Only about 18.1 min/d (2.1%) was spent at ≥ 100 steps/min. Although this value is somewhat higher than that reported for United States adults, with an average of 7 minutes spent in this category, it is still low. In previous empirical studies, 100 steps/min has been shown to correspond to moderate-intensity activity (3 METs) [24,25]. In the public PA guideline, it is recommended to participate in moderate-intensity of PA at least 150 min/wk to promote health [1]. It was found that Korean adults spent about 115 min/wk at a cadence of at least 100 steps/min, which is lower than the general guideline level. Among the 1,703 adults, 546 (32.1%) spent ≥ 150 min/wk at a moderate to vigorous stepping cadence (data not shown). In contrast, based on activity counts using Troiano et al. [26] classification, Lim et al. [22] reported that Korean adults spent about 35 min/d in moderate to vigorous PA, and 56.1% of Korean adults met the PA guideline. Cadence-based measurements show lower adherence than other criteria. Although some studies reported that the “peak cadence”, which refers to the highest cadence in a day, was associated with metabolic risk factors [17,27], to our knowledge, no study has investigated the amount of PA, as defined by various cadence categories, that can provide health benefits.

We examined differences in the cadence pattern according to sex and age. No sex difference was found according to sex for non-movement, but males had higher values for all cadence categories than females except for the incident movement and faster locomotion categories. These results are similar to those of a study of United States adults [15]. Interestingly, as in our study, males spent more time than females in most cadence categories except for incidental movement and faster locomotion. However, there is not enough evidence to explain this pattern. There was no significant difference in the wearing time of the accelerometer according to sex (p= 0.867).

The older age groups were more likely to have less cumulative time and percentage per day at non-movement cadence in both sexes, while the opposite trend was found for United States adults [15]. In addition, older adults (60-64 years) tended to have higher cumulative time and percentage of time at a high cadence level than younger age groups (30-59 years). In general, it is known that older people have higher sedentary time and engage in less moderate to vigorous PA, which is inconsistent with our finding [28,29]. The results of the current study are similar to the results of a previous study that investigated the PA of KNHANES participants through self-reported questionnaires and activity counts [22]. On average, the oldest group walked more per day than the groups aged 30-59, as shown in Table 1. Therefore, it is possible that the older adults who voluntarily participated in this study may have been physically healthy and had active habits.

There are some limitations that must be acknowledged in this study. PA was only classified based on ambulatory behavior measured using an accelerometer; thus, upper body movements or water-based activity without the accelerometer were not reflected in our results. In this study, as the participants wore the accelerometer for only 1 week and some participants only had 1 valid day, there might be a difference from the true cadence pattern. The cadence categories were classified based on empirical research, but the relative intensity of cadence may vary depending on personal characteristics such as height and age [30].

In summary, free-living stepping cadence patterns in Korean adults were analyzed in this study. There was a linear decreasing trend in the cumulative time and proportion across cadence categories. In addition, Korean adults spent most of their time in a day at a low cadence level (< 40 steps/min). They spent only 18.1 minutes (2.1%) in over 100 steps/min. There were differences between sex in the cumulative time and percentage in each cadence category; however, the overall linear trends were consistent. The older age group spent less time in the non-movement cadence category, and the youngest and oldest groups spent more time at a higher cadence (≥ 100 steps/min) than other groups. Furthermore, this pattern was consistent in a subgroup analysis by sex. These results indicate that Korean adults generally engage in low-intensity ambulatory behavior in all sex and age groups. In the future, it will be necessary to examine the dose of cadence-based PA that provides health benefits.

## Figures and Tables

**Table 1.** Demographic characteristics of the participants, stratified by sex and age groups

Characteristics			All (n=1,703)	Sex		Age, yr				
				Male (n=635)	Female (n=1,068)	19-29 (n=313)	30-39 (n=355)	40-49 (n=402)	50-59 (n=452)	60-64 (n=181)
Age (yr)			43.30±0.31	42.93±0.53	43.52±0.38	23.72±0.18	34.91±0.15	44.74±0.14	54.70±0.13	61.95±0.10
Female			1,068 (61.7)	-	-	187 (59.7)	219 (61.7)	264 (65.7)	292 (64.6)	106 (58.6)
Height (cm)			163.37±0.21	171.31±0.24	158.65±0.18	166.36±0.45	166.04±0.45	163.15±0.40	160.80±0.38	159.86±0.62
Weight (kg)			63.28±0.29	72.07±0.43	58.05±0.28	62.53±0.74	65.59±0.71	62.73±0.56	62.85±0.50	62.31±0.76
BMI (kg/m^2^)			23.62±0.08	24.54±0.13	23.07±0.10	22.48±0.21	23.65±0.19	23.48±0.16	24.25±0.15	24.30±0.22
	BMI category (kg/m^2^)									
		Underweight (<18.5)	75 (4.4)	12 (1.9)	63 (5.9)	30 (9.6)	18 (5.1)	15 (3.7)	8 (1.8)	4 (2.2)
		Normal weight (18.5-22.9)	705 (41.4)	205 (32.3)	500 (46.8)	164 (52.4)	149 (42.0)	180 (44.8)	155 (34.3)	57 (31.5)
		Overweight (23.0-24.9)	406 (23.8)	160 (25.2)	246 (23.0)	61 (19.5)	74 (20.8)	99 (24.6)	122 (27.0)	50 (27.6)
		Obese (≥25.0)	517 (30.4)	258 (40.6)	259 (24.2)	58 (18.5)	114 (32.1)	108 (26.9)	167 (36.9)	70 (38.7)
Daily steps (steps/d)^1^			7,968.68±90.22	8,416.51±153.85	7,702.41±110.28^2^	7,659.05±201.17	7,506.58±191.85	8,024.16±187.79	8,161.85±177.51	8,804.83±285.45

Values are expressed as mean (standard error) for continuous variables and sample size (%) for categorical variables.

1 p for trend of age groups (p for trend<0.001).

2 Significantly different from male (p<0.001).

**Table 2.** Spent time (absolute and percentage value) in cadence categories during wearing time by sex

Categories (min/d^1^/%^2^)	All (n=1,703)	Male (n=635)	Female (n=1,068)	p-value
Non-movement	360.08±2.56/43.59±0.30	361.76±4.59/43.75±0.53	359.07±3.03/43.50±0.35	0.625/0.694
Incidental movement	361.50±2.28/43.57±0.24	345.34±3.94/41.57±0.41	371.12±2.73/44.76±0.28	<0.001/<0.001
Sporadic movement	49.26±0.86/5.95±0.10	57.21±1.50/6.91±0.18	44.54±1.02/5.37±0.12	<0.001/<0.001
Purposeful steps	18.86±0.40/2.24±0.05	23.91±0.75/2.87±0.09	15.85±0.44/1.87±0.05	<0.001/<0.001
Slow walking	10.83±0.20/1.25±0.03	13.11±0.39/1.53±0.05	9.47±0.21/1.08±0.03	<0.001/<0.001
Medium walking	9.33±0.18/1.07±0.02	10.31±0.33/1.19±0.04	8.74±0.21/1.00±0.03	<0.001/<0.001
Brisk walking	12.77±0.30/1.48±0.04	12.64±0.51/1.46±0.07	12.85±0.37/1.49±0.05	0.732/0.681
Faster locomotion	5.35±0.22/0.56±0.03	4.20±0.36/0.42±0.05	6.03±0.18/0.64±0.04	<0.001/<0.001

Values are presented as mean±standard error.

1 The mean time across the valid day for each cadence category.

2 The mean percentage of minutes based on monitored total wear time.

**Table 3.** Spent time (absolute and percentage value) in cadence categories during wearing time across age groups

Categories (min/d^1^/%^2^)	Age, yr					p for trend
	19-29 (n=313)	30-39 (n=355)	40-49 (n=402)	50-59 (n=452)	60-64 (n=181)	
Non-movement	399.09±5.89/50.02±0.70	355.38±5.52/43.46±0.62	356.61±5.26/43.16±0.58	341.77±4.72/40.82±0.54	335.26±7.72/40.62±0.91	<0.001/<0.001
Incidental movement	302.22±5.12/37.60±0.57	355.56±4.46/43.60±0.48	374.11±4.35/44.14±0.45	388.85±4.32/46.31±0.42	379.38±6.85/45.72±0.71	<0.001/<0.001
Sporadic movement	40.00±1.70/4.93±0.20	52.67±1.86/6.46±0.23	52.08±1.82/6.16±0.21	50.71±1.72/6.09±0.20	48.74±2.80/5.88±0.33	<0.001/<0.001
Purposeful steps	16.73±0.76/2.02±0.09	19.82±0.91/2.40±0.11	19.49±0.91/2.27±0.10	18.83±0.77/2.22±0.09	19.30±1.28/2.29±0.15	0.137/0.179
Slow walking	10.60±0.44/1.23±0.06	10.63±0.41/1.25±0.05	10.68±0.40/1.17±0.05	10.82±0.39/1.25±0.05	11.97±0.78/1.43±0.10	0.407/0.116
Medium walking	10.41±0.45/1.23±0.06	8.79±0.36/1.01±0.05	8.63±0.36/0.96±0.05	9.18±0.36/1.04±0.05	10.41±0.55/1.20±0.07	<0.001/<0.001
Brisk walking	16.42±0.74/2.00±0.10	10.11±0.57/1.15±0.07	11.36±0.54/1.27±0.07	12.40±0.60/1.42±0.08	15.73±1.07/1.85±0.13	<0.001/<0.001
Faster locomotion	5.59±0.41/0.60±0.06	3.45±0.45/0.33±0.06	5.97±0.49/0.61±0.06	5.49±0.41/0.58±0.05	6.92±0.90/0.76±0.11	0.017/0.024

Values are presented as mean±standard error.

1 The mean time across the valid day for each cadence category.

2 The mean percentage of minutes based on monitored total wear time.
